# Outcomes of internal fixation versus hemiarthroplasty for elderly patients with an undisplaced femoral neck fracture: a systematic review and meta-analysis

**DOI:** 10.1186/s13018-019-1377-5

**Published:** 2019-10-11

**Authors:** Hsuan-Hsiao Ma, Te-Feng Arthur Chou, Shang-Wen Tsai, Cheng-Fong Chen, Po-Kuei Wu, Wei-Ming Chen

**Affiliations:** 1 Department of Orthopaedics and Traumatology, Taipei Veterans General Hospital, No. 201, Sec 2, Shi-Pai Road, Taipei, 112 Taiwan; 2 Department of Orthopaedics, School of Medicine, National Yang-Ming University, Taipei, Taiwan

**Keywords:** Elderly, Femoral neck fracture, Hemiarthroplasty, Hip fracture, Internal fixation, Reoperation

## Abstract

**Background:**

Although internal fixation has been the main treatment option for elderly patients with an undisplaced femoral neck fracture, it is associated with a high reoperation rate. Some surgeons have discussed the use of hemiarthroplasty, but there is limited literature comparing these two treatment modalities. In this study, we compared the perioperative results of hemiarthroplasty with internal fixation for undisplaced femoral neck fractures.

**Methods:**

We performed a comprehensive review of literatures on PubMed, Web of Science, Embase, and the Cochrane Library for randomized controlled trials and comparative observational studies. Of the 441 studies initially identified, 3 met all inclusion criteria. Two reviewers independently graded study quality and abstracted relevant data including reoperation rate, mortality rate, Harris Hip Score (HHS), length of hospital stay, and operation duration.

**Results:**

Our results revealed that hemiarthroplasty was associated with a lower reoperation rate than the internal fixation group (OR 4.489; 95% CI 2.030 to 9.927). Mortality rate at postoperative 1 month and 1 year and HHS at postoperative 1 year and 2 years were not different. Length of hospital stay (SMD − 0.800, 95% CI − 1.011 to − 0.589) and operation duration (SMD − 2.497, 95% CI − 2.801 to − 2.193) were shorter in the internal fixation group.

**Conclusions:**

Compared with the internal fixation group, patients that underwent hemiarthroplasty had a lower reoperation rate and an equivalent overall mortality rate. Our meta-analysis suggests that hemiarthroplasty might be a better treatment choice than internal fixation in treating elderly patients with an undisplaced femoral neck fracture.

## Introduction

In current practice, internal fixation has been the treatment of choice for undisplaced femoral neck fractures. However, most studies have reported a high reoperation rate after internal fixation (ranged from 8 to 34.6%) [1–10]. Therefore, several alternative options have been discussed. One of the most commonly performed surgeries is hemiarthroplasty [11–14]. In current literature, the perioperative outcomes between hemiarthroplasty and internal fixation remain inconclusive with regard to reoperation rate, mortality rate, and functional outcome. Sikand et al. validated that hemiarthroplasty surgery was an independent risk factor for increased 1-month and 1-year mortality [12] while two other studies did not find a difference [11, 13]. In terms of reoperation rate, two studies noted similar results between the two treatment modalities. In contrast, Dolatowski et al. found a lower reoperation rate in the hemiarthroplasty group. On the other hand, improved functional outcome was noted in patients that underwent hemiarthroplasty [11], while results from another study did not reveal a difference [13].

Due to these inconclusive results, we conducted this meta-analysis to evaluate several outcome parameters for elderly patients that underwent either hemiarthroplasty or internal fixation for undisplaced femoral neck fractures. We hypothesize that patients receiving hemiarthroplasty is associated with a lower risk of reoperation and will have improved functional status compared with patients that underwent internal fixation.

## Materials and methods

### Search strategy

**Fig. 1:**
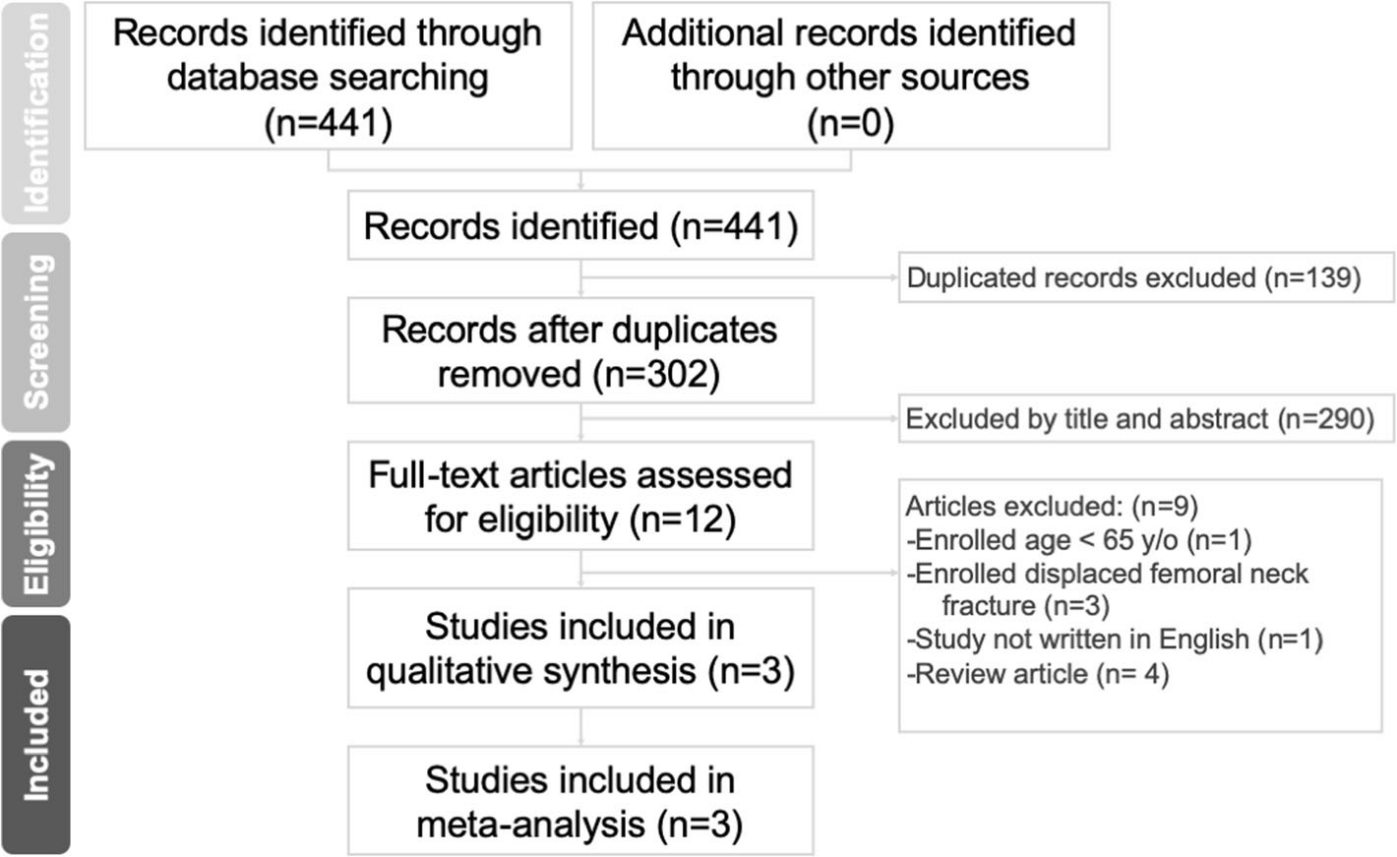
Preferred Reporting Items for Systematic Reviews and Meta-Analysis (PRISMA) flow diagram for the searching and identification of included studies

We conducted a systematic search on PubMed, Web of Science, Embase, and the Cochrane Library to identify relevant studies from the earliest record to May 2019. The bibliographies of the included studies were manually reviewed for relevant references. Studies not written in English or not available in full text were excluded. We investigated studies that compare the outcomes after internal fixation or hemiarthroplasty procedures for elderly patients with undisplaced femoral neck fracture. The search strategy comprised the following keywords in variable combination: (femoral neck fracture) AND (undisplaced OR nondisplaced) OR (internal fixation OR fixation OR hemiarthroplasty OR arthroplasty). Regarding the types of included studies, we enrolled randomized controlled trials (RCTs) and comparative observational studies. Single-armed follow-up studies, case series, and case reports were also excluded. All identified studies were required to comprise two treatment arms, one of which was internal fixation and the other was hemiarthroplasty. The search strategy is presented in Fig. 1.

### Inclusion criteria

We considered studies that were eligible if they met the PICOS criteria (population, intervention, comparator outcomes, study design): population—elderly patients (≥ 65 years old) with undisplaced femoral neck fracture; intervention—internal fixation or hemiarthroplasty as the surgical treatment method for undisplaced femoral neck fracture; comparator—internal fixation or hemiarthroplasty procedure; and outcomes—reoperation rate, 1-month and 1-year mortality rate, Harris Hip Score (HHS) at postoperative 1 year and 2 years, length of hospital stay, and operation duration. Studies must have a follow-up rate of at least 90%, and at least one of the above outcome domains must be included. We only included randomized controlled trials or comparative observational studies.

### Data extraction and quality assessment

Two reviewers examined all the identified articles and extracted data using a predetermined form. We recorded the first author, year, study design, enrolled sample number, sex, age, internal fixation method, outcome domains to assess reoperation rate, 1-month and 1-year mortality rate, HHS at postoperative 1 year and 2 years, length of hospital stay, and operation duration (Table 1). Two reviewers independently evaluated the methodological quality of the enrolled studies using the Cochrane Collaboration to reduce bias and to ensure our results were reliable and veritable. Discrepancies between the two reviewers were solved after thorough discussion.

**Table 1:** Characteristics of included studies

First author, year	Study design	Enrolled sample number (G1/G2)	Sex, female (G1/G2)	Age (G1/G2)	Internal fixation method	Outcome domains						
						a	b	c	d	e	f	g
Dolatowski, 2019 [11]	RCT	111/108	76%/68%	83.2/83.1	Two partially threaded, cancellous, cannulated screws of 8.0-mm diameter	V	V	V	V	V	V	V
Lu, 2017 [13]	RCT	41/37	70.7%/78.4%	85.85/86.2	Three 6.5-mm cannulated screws	V		V	V	V	V	V
Sikand, 2004 [12]	Prospective comparative observational study	110/29	77.2%/72%	77/79	Three 6.5-mm cancellous lag screws (*n* = 104) Dynamic hip screw (DHS, *n* = 6)	V	V	V			V	

*G1* group of internal fixation, *G2* hemiarthroplasty, *RCT* randomized controlled trial. Outcome measurement: *a* reoperation rate, *b* 1-month mortality, *c* 1-year mortality, *d* postoperative 1-year HHS, *e* postoperative 2-year HHS, *f* length of hospital stay, *g* operation duration

### Evaluation of publication bias

**Fig. 2:**
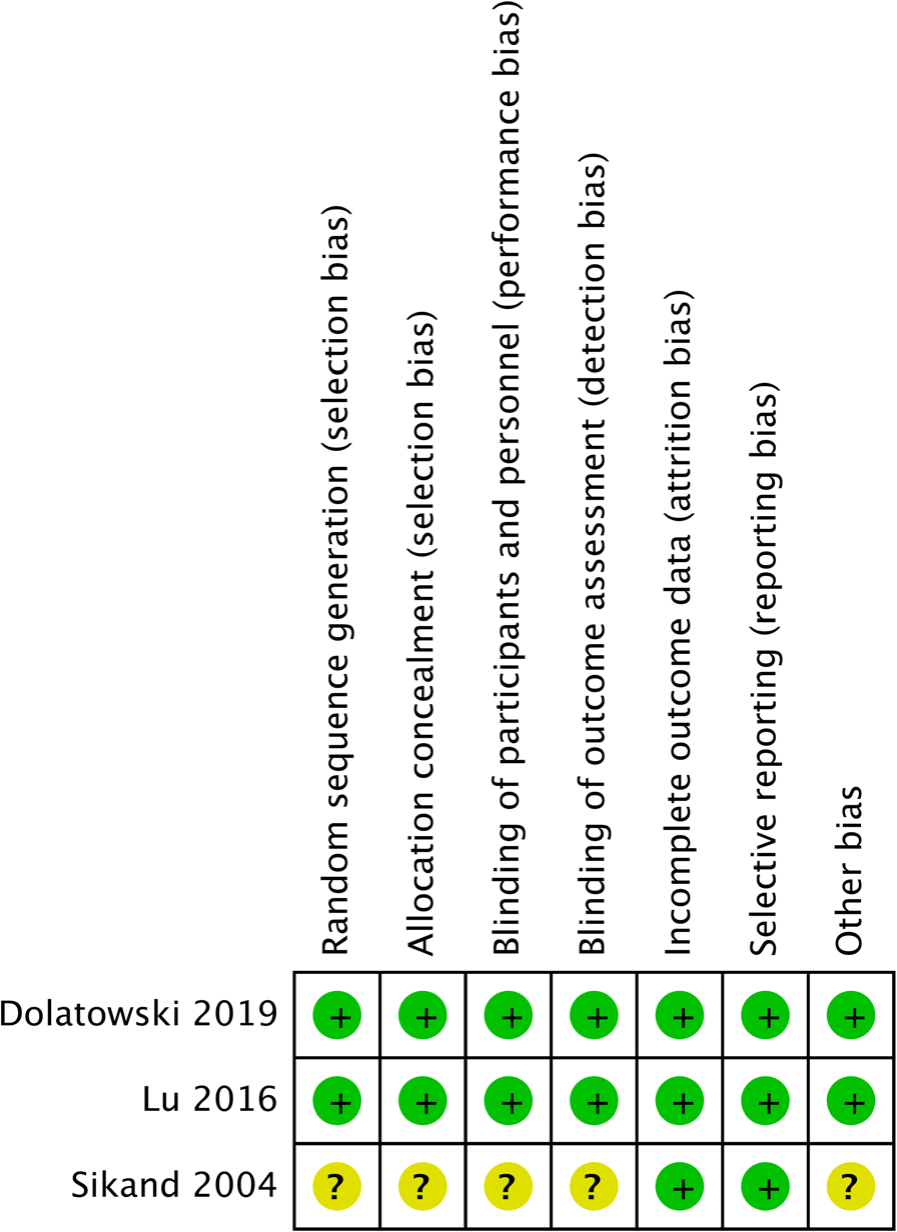
Assessment for the risk of bias

**Fig. 3:**
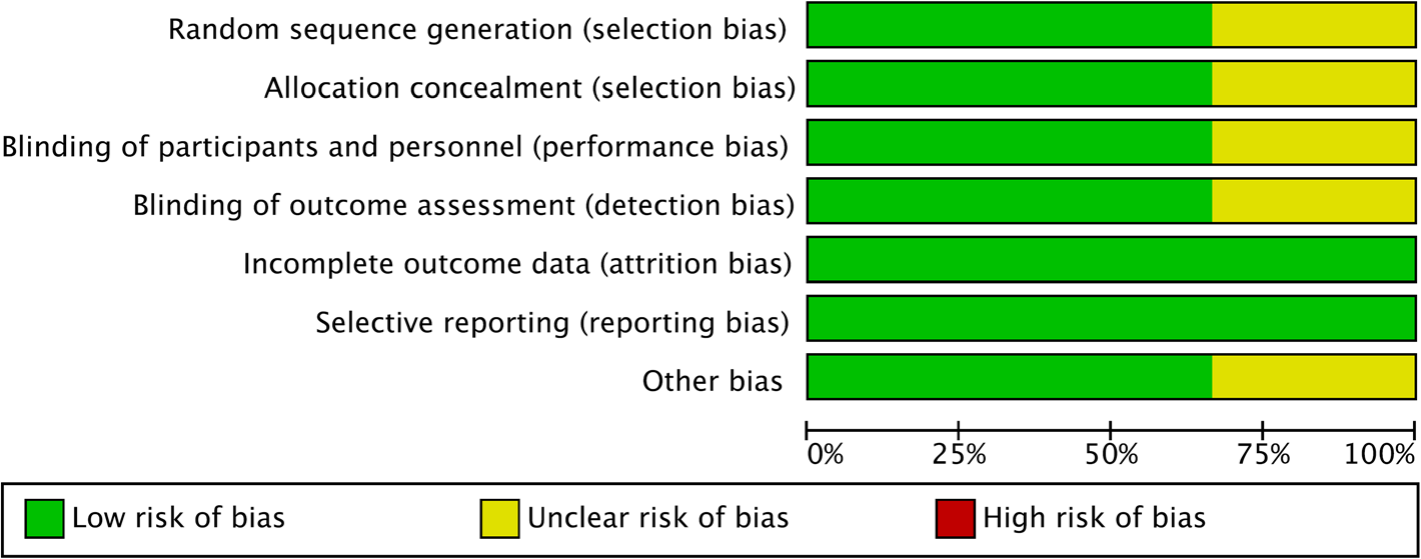
Risk of bias evaluation for each study according to the recommendations of the Cochrane Collaboration

A thorough risk-of-bias assessment was completed to identify factors that may have altered the results of this analysis. Two senior reviewers independently evaluated each included study and documented their potential for selection bias, performance bias, detection bias, attrition bias, and reporting bias using the Cochrane tool for assessing risk of bias of the enrolled studies (Figs. 2 and 3).

### Data synthesis

The odds ratio (ORs) of the reoperation rate and 1-month and 1-year mortality rate between the internal fixation and hemiarthroplasty group were the primary outcome. The standardized mean differences (SMDs) of Harris Hip Score (HHS) at postoperative 1 year and 2 years, length of hospital stay, and operation duration were the secondary outcome. A negative SMD value or OR value less than 1 indicated that internal fixation is a favorable treatment option. A random effects model was utilized to pool individual SMDs and ORs. Analyses were performed using Comprehensive Meta-Analysis (CMA) software, version 3 (Biostat, Englewood, NJ, USA). Between-trial heterogeneity was determined by using *I*^2^ tests; values > 50% were regarded as considerable heterogeneity. Statistical significance was defined as *p* values < 0.05.

## Results

### Search results

We identified 441 relevant articles according to the search strategy. One hundred thirty-nine duplicate records were removed using Endnote software. Two hundred ninety were excluded after reading the title and abstract. According to the inclusion criteria, 9 studies were excluded after reading the full article. Finally, 3 articles that compared internal fixation and hemiarthroplasty in undisplaced femoral neck fracture were included for our meta-analysis. The baseline characteristics of the 3 included studies are summarized in Table 1. Two of them were randomized controlled trials, and the other was a prospective observational study.

### Meta-analysis results

#### Reoperation rate

**Fig. 4:**
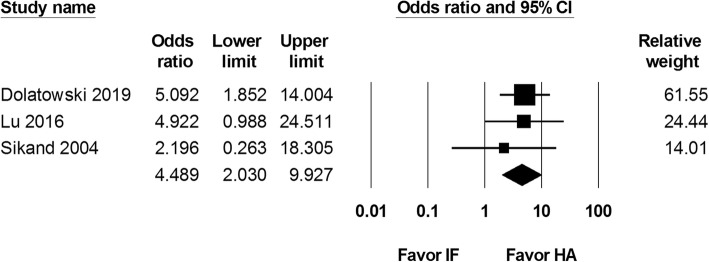
Forest plot comparing reoperation rate after internal fixation (IF) versus hemiarthroplasty (HA)

Three studies reported the reoperation rates after internal fixation and hemiarthroplasty surgery. A total of 262 internal fixation and 174 hemiarthroplasty procedures were completed. Our results revealed a higher reoperation rate after internal fixation than after hemiarthroplasty with an odds ratio of 4.489 (95% CI 2.030 to 9.927; Fig. 4).

#### One-month and 1-year mortality rate

**Fig. 5:**
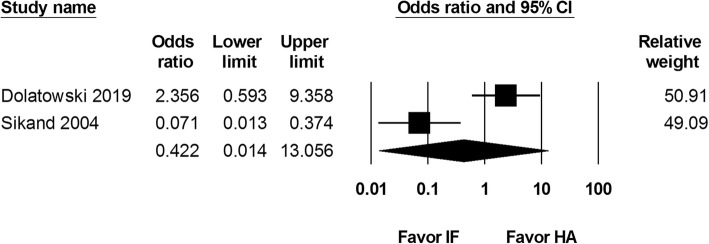
Forest plot comparing 1-month mortality rate after internal fixation (IF) versus hemiarthroplasty (HA)

**Fig. 6:**
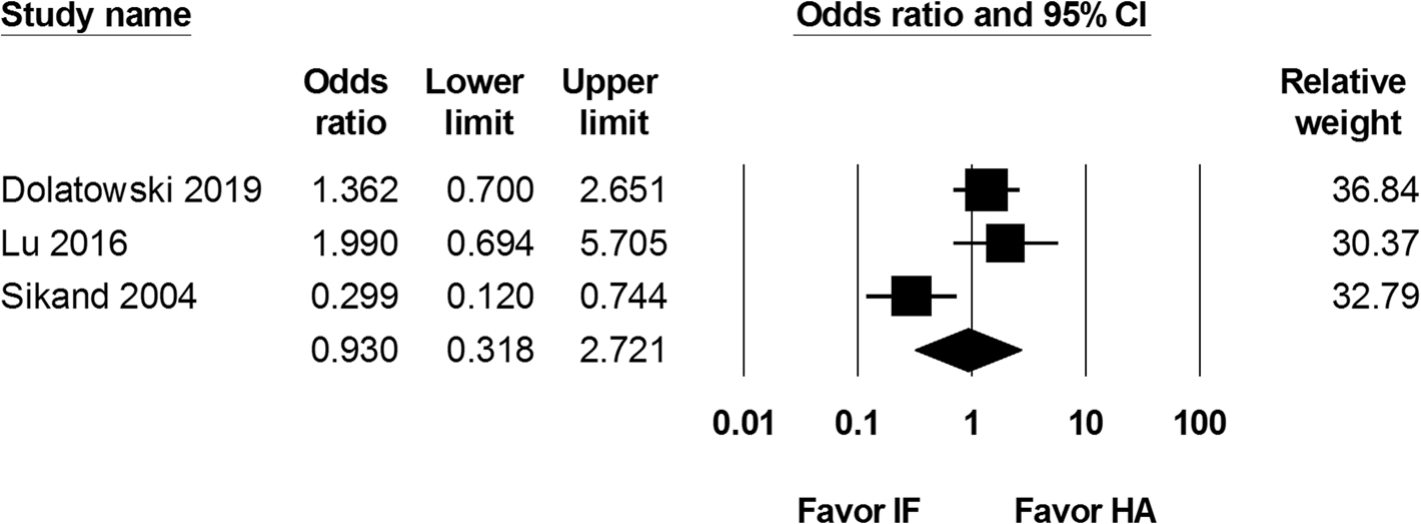
Forest plot comparing 1-year mortality rate after internal fixation (IF) versus hemiarthroplasty (HA)

We included all-cause mortality reported within the first month and the first year after the procedure. Two studies that reported 1-month mortality rate were included, with 221 internal fixation and 137 hemiarthroplasty procedures. Data from these two studies showed an odds ratio of 0.422 (95% CI 0.014 to 13.056; Fig. 5). Three studies identified the 1-year mortality rate (262 internal fixation and 174 hemiarthroplasty procedure). There was no significant difference in 1-year mortality rate between the two groups (OR 0.930, 95% CI 0.318 to 2.721; Fig. 6).

#### Harris Hip Score at postoperative 1 year and 2 years

**Fig. 7:**
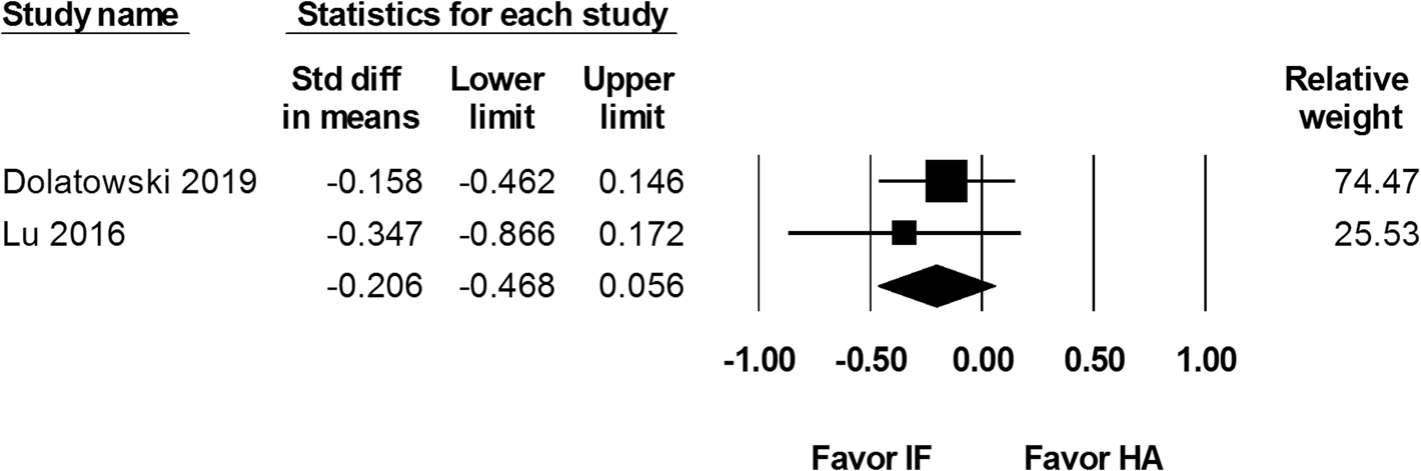
Forest plot comparing Harris Hip Score at postoperative 1 year after internal fixation (IF) versus hemiarthroplasty (HA)

**Fig. 8:**
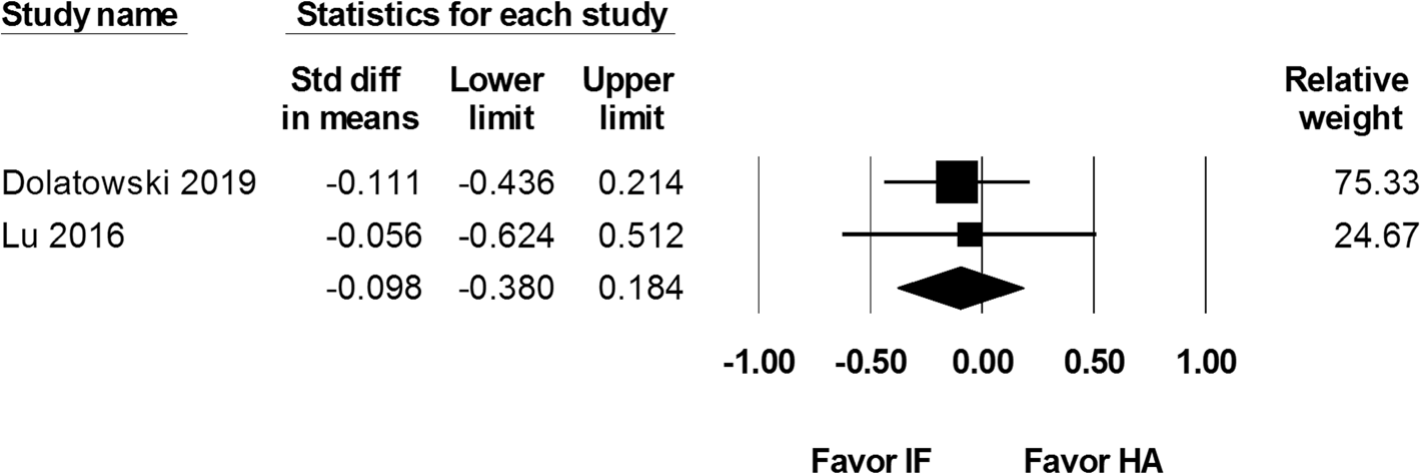
Forest plot comparing Harris Hip Score at postoperative 2 years after internal fixation (IF) versus hemiarthroplasty (HA)

Two studies reported Harris Hip Score at 1 year after 111 internal fixation and 114 hemiarthroplasty procedures. The results showed an overall SMD of − 0.206 (95% CI − 0.468 to 0.056; Fig. 7). Two studies including 92 internal fixation and 102 hemiarthroplasty procedures reported Harris Hip Score at postoperative 2 years. The results showed no difference between the two groups (SMD − 0.098, 95% CI − 0.380 to 0.184; Fig. 8).

#### Length of hospital stay

**Fig. 9:**
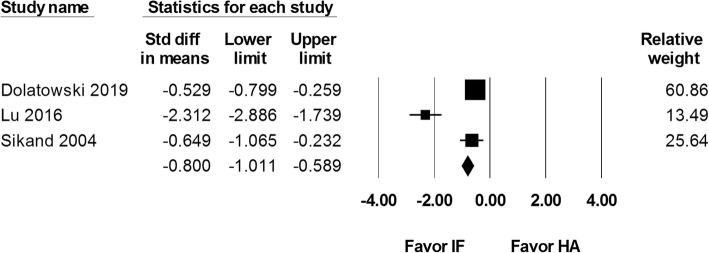
Forest plot comparing length of hospital stay after internal fixation (IF) versus hemiarthroplasty (HA)

Length of hospital stay was reported in all three studies. Data were included from 262 internal fixation and 173 hemiarthroplasty procedures. The analysis reported a significantly shorter hospital stay after internal fixation than hemiarthroplasty (SMD − 0.800, 95% CI − 1.011 to − 0.589; Fig. 9).

#### Operation duration

**Fig. 10:**
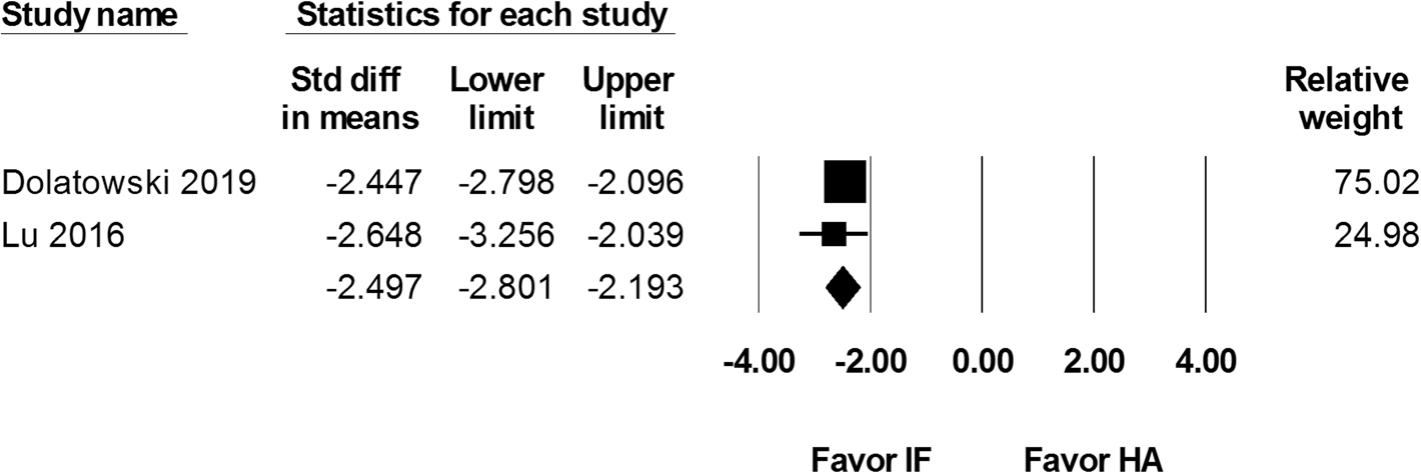
Forest plot comparing operation duration after internal fixation (IF) versus hemiarthroplasty (HA)

Two studies involving 151 internal fixation and 145 hemiarthroplasty procedures reported results for operation duration, which was recorded in minutes. There was a significantly shorter operation duration for patients that underwent internal fixation (SMD − 2.497, 95% CI − 2.801 to − 2.193; Fig. 10).

## Discussion

In this meta-analysis, we evaluated the outcome of elderly patients with undisplaced femoral neck fractures that underwent internal fixation or hemiarthroplasty. This study provides a synthesis of evidence from two randomized controlled trials and one prospective observational study to determine the optimal procedure in dealing with elderly patients presenting with undisplaced femoral neck fracture.

Our primary outcome comprised of reoperation rate and 1-month and 1-year mortality rate. Our analysis revealed a significantly higher reoperation rate in the internal fixation group (OR 4.489, 95% CI 2.030 to 9.927). One-month and 1-year mortality rate was equivalent between internal fixation and hemiarthroplasty. Secondary outcome included Harris Hip Score at postoperative 1 year and 2 years, length of hospital stay, and operation duration. The internal fixation group was associated with a shorter length of hospital stay (SMD − 0.800, 95% CI − 1.011 to − 0.589) and a shorter operation duration (SMD − 2.497, − 2.801 to − 2.193). Harris Hip Score at postoperative 1 year and 2 years was not different.

In current literature, the treatment of choice for undisplaced femoral neck fractures is with internal fixation. However, there is a high reoperation rate (8 to 34.6% [1–10]), which has led to several alternative options being proposed. Several complications such as loss of fixation, nonunion, and avascular necrosis are the most common reasons for reoperation. A subsequent conversion to hip arthroplasty might be required to restore function and relieve pain. The reported rate of conversion to hip arthroplasty ranged from 8 to 16% [1, 5, 9]. In a prospective case series of 383 patients that received internal fixation for garden type I or II femoral neck fractures, 10% of patients had a salvage arthroplasty. It was estimated that up to one fourth of long-term survivors needed a conversion to arthroplasty [5]. Therefore, several studies have been conducted to validate hip arthroplasty as a possible alternative treatment for undisplaced femoral neck fracture [11–14].

According to current literature, we defined “elderly” patients as a chronological age of 65 years or older [15]. Based on this definition, there was one prospective observational study and two randomized controlled trials that met our inclusion criteria. Sikand et al. enrolled 139 elderly patients with undisplaced femoral neck fracture surgically treated with internal fixation (*N* = 110) or hemiarthroplasty (*N* = 29). The authors noted a shorter operation duration and length of hospital stay in the internal fixation group. The reoperation rate was not significantly different between internal fixation (*N* = 8 of 110, 7.2%) and hemiarthroplasty (*N* = 1 of 29, 3%). However, patients that had undergone hemiarthroplasty surgery had a higher 1-month (21% vs. 2%) and 1-year (38% vs. 16%) mortality rate. In addition, pre-injury residential status was also a significant factor that influenced early mortality. Therefore, the authors concluded that they did not recommend hemiarthroplasty as the initial treatment option for undisplaced femoral neck fractures [12]. However, the functional outcome for these patients was not assessed in this study. Two randomized controlled trials were conducted to compare the outcome between internal fixation and hemiarthroplasty, including reoperation rate, mortality rate, and functional scores. Lu et al. [13] conducted a randomized controlled trial including 78 patients over 80 years of age that were treated with internal fixation (*N* = 41) or hemiarthroplasty (*N* = 37) for undisplaced femoral neck fractures. In patients that received internal fixation, there was a shorter length of surgical incision, operation duration, and length of hospital stay. There were also less blood loss and lower transfusion rates. There was a trend toward a higher reoperation rate in the internal fixation group (9/41, 22%) versus hemiarthroplasty group (2/37, 5.4%, *p* = 0.051). In contrast to the results from Sikand et al. [12], patient cumulative survival rates were similar between the two treatment groups. The Harris Hip Score at postoperative 12, 24, 36, 48, and 60 months was assessed, but there was no significant difference between the two groups [16]. In a multicenter randomized controlled trial conducted by Dolatowski et al. [11], 219 patients ≥ 70 years old with a nondisplaced femoral neck fracture received either internal fixation (*N* = 111) or hemiarthroplasty (*N* = 108). The Harris Hip Score (HHS) and other functional outcome domains including the timed “Up & Go” (TUG) test, pain intensity numerical rating scale (PI-NRS), EuroQol-5 Dimension-3 level scale (EQ-5D), and mini-mental state examination (MMSE) were assessed at prefracture and postoperative 3 months, 12 months, and 24 months [16–21]. The patients in the hemiarthroplasty group demonstrated a significantly improved mobility (TUG test) in postoperative 12 and 24 months. The HHS, PI-NRS, and MMSE were similar at all time points between the two groups. For patients that received internal fixation, a shorter operation duration and length of hospital stay and less intraoperative blood loss were noted. There was a lower major operation rate (hemiarthroplasty vs. internal fixation, 5% vs. 20%, *p* = 0.002) and combined major and minor reoperation rate (hemiarthroplasty vs. internal fixation, 7.4% vs. 24.3%, *p* < 0.05) in the hemiarthroplasty group. Mortality rate was not different at postoperative 3, 12, and 24 months. The authors found hemiarthroplasty to be superior to internal fixation with regard to a lower major reoperation rate and improved function outcome as assessed by the TUG test. We conducted this meta-analysis because of the inconclusive results with regard to outcome domains including mortality rate, reoperation rate, and functional outcomes. Our analysis revealed that hemiarthroplasty for undisplaced femoral neck fracture in the elderly patients might be a viable treatment option compared with internal fixation in terms of a lower reoperation rate and an equivalent mortality rate.

The reasons for reoperation after an internal fixation or hemiarthroplasty surgery for an undisplaced femoral neck fracture are quite distinct from each other. In patients who had undergone internal fixation, the most common causes include fixation failure, nonunion, and osteonecrosis. A conversion to hip arthroplasty is usually necessary to restore mobility and relieve pain [1, 3, 5]. Several other reasons such as periimplant fracture and hardware irritation that required a revision surgery (fracture fixation and removal of implant, respectively) are other reasons that may result in additional surgeries [1, 3–5, 9, 11–13, 22]. For patients that received hemiarthroplasty, periprosthetic joint infection, dislocation, and prosthesis loosening were the most common causes of reoperation [11, 13]. In patients with periprosthetic infection, debridement and/or exchange arthroplasty may be required. For patients with recurrent hip dislocations or loosening of prosthesis, a revision surgery is often required [11–13].

In addition to the medical benefits associated with hemiarthroplasty surgery (lower risk of reoperation etc.), the quality of life and healthcare-related costs are also important outcome domains that should be assessed. Dolatowski et al. conducted the only study that compared quality of life between the two groups using the EQ-5D index. The authors noted a higher EQ-5D index in the HA group 2 weeks before fracture and remained proportionate throughout the study [11]. Further studies are required to clarify whether this postoperative difference resulted from the type of surgery, preoperative status, or patient characteristics. There were several randomized controlled trials comparing total costs between hip arthroplasty and internal fixation for displaced femoral neck fracture in the first 1 or 2 years after the surgery [23–25]. One study found similar total costs between internal fixation and hip arthroplasty when secondary surgeries were included [25], while results from other studies revealed that internal fixation was associated with higher total costs [23, 24]. Frihagen et al. reported a lower average cost for initial in-hospital stay (€9044 vs. €11,887, *p* < 0.01) but a subtle higher average total cost (€47,186 vs. €38,165, *p* = 0.09) in the internal fixation group [23]. The lower initial average cost in the internal fixation group was outweighed by the subsequent costs resulted from a higher reoperation rate. However, there is lack of similar study in patients with an undisplaced femoral neck fracture. Further study concerning average total cost, cost per quality-adjusted life year (QALY), disability-adjusted life year (DALY), and life years (LY) gained in patients with an undisplaced femoral neck fracture would be necessary to strengthen the conclusion of an ideal treatment choice.

This study is currently the first meta-analysis to compare the outcome between internal fixation and hemiarthroplasty for elderly patients with an undisplaced femoral neck fracture. However, there are several limitations that should be recognized. First, we searched only for English articles but not articles in other languages or unpublished data. This could be potential source of publication bias. Second, heterogeneity of clinical setting between studies including age, sex, medical comorbidities, internal fixation methods, surgical approaches, and types of implants for hemiarthroplasty should be recognized. Third, we were not able to analyze several outcome domains with clinical importance such as estimated blood loss, drop in hemoglobin, transfusion rate, health-related quality of life, and healthcare direct or indirect costs because of the limited literature.

## Conclusions

The present meta-analysis revealed that hemiarthroplasty led to a lower reoperation rate compared with that of internal fixation. Mortality rate and functional outcome were not different. The findings suggest that hemiarthroplasty might be a better choice than internal fixation in treating elderly patients with an undisplaced femoral neck fracture.

## Data Availability

The datasets used and/or analyzed during the current study are available from the corresponding author on reasonable request.

## Abbreviations

CI: Confidence interval
DALY: Disability-adjusted life year
EQ-5D: EuroQol-5 dimension-3 level scale
HHS: Harris Hip Score
LY: Life years
MMSE: Mini-mental state examination
OR: Odds ratio
PI-NRS: Pain intensity numerical rating scale
QALY: Quality-adjusted life year
RCT: Randomized controlled trial
SMD: Standardized mean difference
TUG: Timed “Up & Go” test
